# The write algorithm: promoting responsible artificial intelligence usage and accountability in academic writing

**DOI:** 10.1186/s12916-023-03039-7

**Published:** 2023-09-04

**Authors:** Steven Bell

**Affiliations:** 1 Precision Breast Cancer Institute, Department of Oncology, University of Cambridge, Cambridge, UK; 2 Cancer Research UK Cambridge Centre, Li Ka Shing Centre, University of Cambridge, Cambridge, UK

Recent strides in large language models, powered by sophisticated artificial intelligence (AI) algorithms trained on extensive language datasets, have revolutionised writing tools [1]. *OpenAI*’s ChatGPT, a leading example, excels at analysing text and generating content based on user input. These breakthroughs have profound implications for academic writing, attracting the attention of journals worldwide [2]. While the pros and cons of adopting these technologies have been extensively debated, the responsible implementation and transparent documentation of their use remain relatively overlooked. This Editorial seeks to fill this gap.

## Potential of large language models in academic writing

Large language models offer immense potential in academic writing. They effectively process vast amounts of data, generating innovative ideas and even entire scholarly manuscripts. Through emulating human writing styles, they have the potential to enhance precision, authority, and emotional connection. These tools improve writing efficiency, allowing more focus on data analysis, and promote inclusivity by assisting authors with language barriers and providing translations for a wider audience. Integrating AI into academic writing can advance knowledge and foster a more interconnected and collaborative academic landscape, but ethical considerations are essential.

## Recognising the current limitations of large language models

Acknowledging the limitations of large language models in human-like authorship is vital. These models rely on data compression, resulting in estimations and compilations rather than precise reproductions, which can lead to the generation of fabricated (“hallucinated”) data [3]. They may also struggle to capture nuanced and specialised knowledge essential for accurate academic writing, similar to how foreign language learners misinterpret idiomatic expressions. It is worth noting that the vast databases large language models are trained on include text based on falsehoods and other sources of untruth [1]. The inability to update training data in real-time also poses concerns regarding the potential inclusion of outdated information [4], particularly in rapidly evolving fields. Hence, exercising caution when considering outputs from these models is crucial, as they may encompass inaccuracies, fabrications (e.g. erroneous references), and potential instances of plagiarism [5]. Ethical concerns are paramount in AI content generation [5]. Large language models may inadvertently perpetuate biases, mirroring skewed representations of certain topics or populations [6]. Failing to evaluate AI text critically risks inaccuracies in scholarly publications, like blindly trusting an inaccurate compass.

Over-reliance on AI without thorough review compromises scholarly rigour. Harnessing AI’s potential as aids to human intellect ensures they complement expertise and discernment rather than replace it.

## Promoting ethical disclosure and responsible use of AI-assisted technologies in academic writing

To address concerns of unethical use of AI in academic writing, researchers are actively developing remedies and detection methods. Additionally, academic journals are implementing processes to ensure transparent AI usage disclosure [7]. While countermeasures are essential to mitigate unethical practices, the responsibility ultimately lies with authors to uphold the highest standards of integrity and ethics in their use of AI tools. By remaining diligent and transparent about AI implementation, researchers can contribute to a more responsible and trustworthy academic landscape, ensuring that AI’s potential benefits are harnessed while minimising its risks.

However, as AI tools become increasingly integrated into the writing process, researchers must navigate new ethical considerations, and the issue of their usage and the required level of disclosure becomes increasingly urgent. For example, many of us have relied on the use of basic AI tools, such as grammar and spelling correction features commonly found in modern word-processing software, for years without disclosure. These commonplace AI utilities are integral to enhancing writing quality. While tools like ChatGPT can be used for similar purposes it is important to acknowledge that they are far more advanced and may also impact the substantive content of the article during the editing process. As a result, their use could warrant disclosure to ensure transparency and ethical reporting [8]. Similarly, when seeking feedback or collaborating on manuscripts, it may become necessary to inquire whether collaborators have used such tools to edit or generate any text, ensuring proper disclosure and compliance with reporting policies.

Less-discussed is the “grey area” of AI-assisted review, where permission to use AI tools on others’ work could be debated. Risks emerge when unpublished content like manuscripts or grants are uploaded for AI processing. This could expose sensitive data and even incorporate unfit findings into training datasets, perpetuating misinformation. Ensuring confidentiality and fair assessment calls for avoiding use of AI tools on unpublished content undergoing peer-review. Instead, rigorous adherence to traditional peer review is recommended. The US National Institutes of Health has issued similar guidance, barring reviewers from using AI tech for assessments [9]. Such policies may serve to mitigate the risk of the echo chamber effect, where biases in AI-assistance tools might go unnoticed if reviewers rely on the same tools for evaluation (Table 1).

**Table 1:** Hypothetical scenario of some potential ramifications following the naïve use of AI-assistance technologies in the research cycle

In this scenario, we encounter Professor Nigel Eve, a distinguished medical doctor with limited knowledge of genomics, delving into the intricacies of breast cancer research using AI-generated content. Employing a large language model AI tool, he embarks on a comprehensive literature review to explore the genetic variants associated with breast cancer
The AI model efficiently presents Professor Eve with a list of seemingly relevant genetic variations linked to breast cancer. Not seeking counsel from a genomics expert or conducting further validation, he incorporates the AI-generated findings into his research paper
However, unbeknownst to him, the AI model’s training data contains incomplete as well as potentially biased information about the genetic variants, leading to the inclusion of inaccurate and misleading details about the relationship between breast cancer and genetics in his paper
Throughout the peer review process, the reviewers, similarly relying on large language models to carry out their duties, reach the same erroneous conclusion as Professor Eve, and the paper is eventually accepted for publication
Trusting Professor Eve’s esteemed reputation as a medical doctor, readers may inadvertently accept these flawed findings, potentially steering other researchers or clinicians toward misguided avenues in their own breast cancer research
Adding to the complexity, one of the genetic variants included in Professor Eve’s research has since been disproven by the scientific community. This critical oversight, stemming from his lack of genomics expertise and failure to perform necessary due diligence, casts doubt on the credibility of his breast cancer research and may have far-reaching consequences
The ramifications of such inaccuracies could impact fellow academics and scientists, leading to misallocation of funding and valuable research time based on erroneous conclusions. Patients, too, may be affected by misguided treatment approaches inspired by this flawed research
In conclusion, Professor Eve’s expedition underscores the paramount importance of amalgamating AI tools with human domain expertise and meticulous due diligence to ensure the accuracy and integrity of research findings. Neglecting these vital steps may lead to misguided scientific pursuits, wasted resources, and, most significantly, potential harm to patients

However, this is not to say that AI-assisted technologies have no utility in providing feedback on others’ work. For example, a researcher may write a rough draft of their thoughts and then “polish” this using a large-language model for clarity and structure. Alternatively, a reviewer could provide a straightforward evaluation and request the tone to be transformed into one of constructive criticism before sending it back to the recipient. Here, one still needs to bear in mind confidentially and, as such, refrain from including identifiable or sensitive data. It is helpful to consider whether you would be displeased by someone revealing similar information about you to a stranger or if sharing such data could in any way harm the individuals being evaluated - now or in the future. If the answer is “yes” to either, then it is best not to share that information with AI tools.

Authors, reviewers, and journal editors share the responsibility of maintaining the highest standards of ethical conduct in the peer review process. As the scientific community embraces the integration of AI technologies, it is essential to balance the advantages of efficiency and accuracy with the ethical considerations surrounding confidentiality and privacy [10].

## Transparency in use of AI-assistance tools

To address these challenges, the International Committee of Medical Journal Editors (ICMJE), which is endorsed by Springer Nature—publisher of *BMC Medicine*—mandates comprehensive disclosure of AI technology usage in all submitted manuscripts (Table 2). It is import to underscore that AI technologies cannot be acknowledged or credited as authors of articles [7, 8], as they do not fulfil the fundamental ICMJE authorship criteria, which encompass the responsibilities of taking ownership for the published work, declaring potential competing interests, and participating in copyright and licensing agreements. Instead, the onus falls on the human authors to fully assume responsibility for ensuring the accuracy, authenticity, and integrity of all AI-generated content. Citing AI-assisted technologies as primary sources should also be avoided. Adherence to these guidelines will preserve scholarly integrity and scientific rigour in academic publishing.

**Table 2:** Requirements for reporting the use of AI-assisted technologies based on ICMJE recommendations

1. Authors must disclose any use of AI-assisted technologies (e.g. large language models, chatbots, image creators) in the creation of submitted work, both in the manuscript and the cover letter
2. AI technologies cannot be listed as authors or co-authors of manuscripts
3. AI technologies cannot be cited as references or primary sources in manuscripts
4. Human authors are responsible for the correctness, completeness, and accuracy of submitted material incorporating AI-assisted technologies
5. Authors must assert the absence of plagiarism in the article, including text and images produced by AI-assisted technologies, and ensure proper attribution of all material, including full citations where appropriate
6. Peer reviewers must refrain from uploading manuscripts to AI software or other technologies that may compromise confidentiality
7. If reviewers choose to use AI technologies to facilitate their reviews, they must disclose the nature and extent of their usage and are responsible for ensuring the accuracy, completeness, and impartiality of any AI-generated content integrated into their reviews

## Conclusions

The updated ICMJE recommendations [7] thoughtfully address AI’s potential consequences in scholarly publishing. Adoption must proceed cautiously, considering limitations in generating misinformation. Transparency, accountability, and ethical use should guide the development and integration of AI-assisted technologies. While AI can assist in various processes, human creativity, curiosity, and ingenuity remain distinctive and invaluable qualities in science and scholarship that will serve as the bedrock of these disciplines for years to come.

## Data Availability

Not applicable.

## Abbreviations

AI: Artificial Intelligence
ICMJE: International Committee of Medical Journal Editors
